# AI-Guided Binding Mechanisms and Molecular Dynamics for MERS-CoV

**DOI:** 10.3390/ijms27041989

**Published:** 2026-02-19

**Authors:** Pradyumna Kumar, Lingtao Chen, Rachel Yuanbao Chen, Yin Chen, Seyedamin Pouriyeh, Progyateg Chakma, Abdur Rahman Mohd Abul Basher, Yixin Xie

**Affiliations:** 1 College of Computing and Software Engineering, Kennesaw State University, Marietta, GA 30060, USA; pkumar7@students.kennesaw.edu (P.K.); lchen25@students.kennesaw.edu (L.C.); spouriye@kennesaw.edu (S.P.); amohdabu@kennesaw.edu (A.R.M.A.B.); 2 Department of Pharmacology and Toxicology, School of Pharmacy, University of Arizona, Tucson, AZ 85721, USA; rachelchen08@arizona.edu (R.Y.C.); bosschen@arizona.edu (Y.C.); 3 Department of Chemistry and Biochemistry, Kennesaw State University, Kennesaw, GA 30144, USA; pchakma@kennesaw.edu

**Keywords:** MERS-CoV, coronavirus, molecular simulations, high performance computing (HPC), salt bridge analysis, hydrogen bonds, binding affinity, drug target, protein–protein interaction (PPI), Artificial Intelligence (AI)

## Abstract

The MERS-CoV (Middle East respiratory syndrome coronavirus) is a zoonotic virus with a high mortality rate and a lack of antiviral drugs, underscoring the need for effective therapeutic methods. Viral entry depends on interactions between viral surface proteins and human receptors, with Dipeptidyl Peptidase-4 (DPP4), a transmembrane glycoprotein, acting as the receptor for MERS-CoV. We employed Molecular Dynamics (MD) Simulations to identify critical interface residues under a high-performance computing (HPC) workflow for accelerated results. Target residue pairs were identified through analysis of salt bridge and hydrogen bond occupancy. The stability of these residues was confirmed through three independent MD Simulations at human body temperature and constant pressure. Additionally, binding affinity predictions were calculated to determine the interaction strength between the virus and human receptors. Applying the scientific threshold criteria, we narrowed our results to seven key interaction pairs; two of the identified pairs (Asp510-Arg317, and Arg511-Asp393) are consistent with findings published in previous research studies, and five novel interactions are proposed for future experimental studies with our active collaborators in Pharmacology. The results provide a molecular basis for targeted mutation-based experiments and support the rational design of structure-based inhibitors aimed at disrupting the MERS-CoV-DPP4 complex, thereby facilitating the translation of computational findings into antiviral drug discovery.

## 1. Introduction

The Middle East Respiratory Syndrome (MERS) is a severe viral respiratory illness caused by MERS Coronavirus (MERS-CoV), with the first outbreak in Saudi Arabia in 2012 [1]. MERS-CoV is classified as a zoonotic pathogen, with dromedary camels identified as the primary animal reservoir responsible for cross-species transmission events into the human population, which translates to a global public health concern [2,3,4]. The World Health Organization (WHO) has reported over 2600 cases with a high case fatality rate of 35%, primarily from the Middle Eastern regions where frequent camel-to-human transmission events occur [1]. Despite being discovered over a decade ago, no licensed antiviral therapeutics or prophylactic vaccines are currently available for MERS-CoV. This persistent translational gap highlights the critical necessity for continued research to elucidate the fundamental molecular mechanisms that govern MERS-CoV infection. Specifically, characterizing the virus’s interaction with host cell receptors is required to provide the structural foundation essential for the rational structure-based design of effective countermeasures [5,6].

The MERS-CoV is part of the betacoronavirus genus, specifically lineage C, and is characterized by its spike glycoprotein (S protein), which mediates cellular entry by binding to host receptors [5]. The receptor-binding domain (RBD) of the MERS-CoV spike protein specifically targets Dipeptidyl Peptidase-4 (DPP4) as its primary functional receptor [7]. DPP4, also known as CD26, is a ubiquitous transmembrane glycoprotein with established roles in both immune regulation and glucose metabolism [8]. Its presence on host cells facilitates MERS-CoV binding and subsequent viral entry [9]. The molecular complex considered in this study involves the MERS-CoV spike protein receptor-binding domain (RBD) and the human DPP4, which provides a foundational experimental structure for effectively studying the protein–protein interactions (PPI) between the virus and host [5,7,9]. The significance of protein–protein interactions (PPIs), such as those between the MERS-CoV RBD and DPP4, lies in their pivotal role in viral pathogenesis and their potential as targets for drug development [10].

Structural and biochemical studies by Wang and colleagues [5] established that the RBD, situated within the S1 subunit of the viral spike glycoprotein, directly engages with the β-propeller domain of host DPP4 [11,12,13]. Experimental evidence further confirms that the MERS-CoV S1 subunit, specifically amino acids 367–606, contains this domain. The RBD mediates the critical initial attachment to human DPP4, facilitating subsequent membrane fusion between the viral envelope and the host cell membrane [14]. Given that this specific interface is essential for viral entry, the MERS-CoV RBD is a prominent and validated therapeutic target for the development of neutralizing antibodies and specific inhibitors aimed at blocking viral attachment [15].

Over the years, the understanding of MERS-CoV, SARS-CoV-2, and SARS-CoV-like infection and their potential for drug discovery has been shaped by the integration of both experimental and computational biology approaches [16,17]. The experimental studies indicate the distinctive features of the spike protein structure by identifying neutralizing antibodies based on viral entry points from RBD [5,11]. Concurrently, computational methods, such as molecular dynamics (MD) simulations and molecular docking, have provided essential mechanistic and dynamic insights into conformational changes, receptor binding kinetics, and the precise identification of drug target areas on the MERS-CoV spike protein [18,19]. These computational approaches accelerate the discovery of small-molecule inhibitors capable of disrupting RBD-DPP4 interactions to effectively block viral entry [20]. Therefore, the successful development of effective countermeasures against emerging viruses is contingent upon the synergistic integration of experimental validation and advanced computational modeling to bridge the gap between structural insights and translational drug discovery.

To support the development of structure-based therapeutics, we leverage computational methods to design molecular models for the structure-based drug discovery against MERS-CoV. Building upon our previous comparative analyses of SARS-CoV-2 and SARS-CoV spike RBD interactions with human ACE2 (Angiotensin-converting enzyme 2) [21,22], the current study utilizes an optimized computational workflow to investigate the residual interactions within the MERS-CoV RBD-DPP4 complex. We employed the MD simulation approach using a High-Performance Computing (HPC) workflow to thoroughly characterize the dynamic protein–protein interactions (PPIs) [23]. The simulations were executed using the OpenMM Python toolkit on a GPU-accelerated system [24]. To ensure the statistical integrity and robustness of our observations, three independent MD simulations were performed under identical configurations and physiological conditions [18].

Following the MD simulations, we quantified key non-covalent interactions, analyzing salt bridge distances and hydrogen bond occupancies to pinpoint critical interface residue pairs governing receptor binding. Analysis was performed using ChimeraX (v1.9) [25], and the mechanistic findings were quantitatively supported by binding free energy predictions utilizing our published computational tool [26]. Our analysis concluded with the identification of seven critical residue pairs governing MERS-CoV attachment to DPP4. Notably, two of these identified pairs (Asp510-Arg317, and Arg511-Asp393) directly align with the key salt bridge interactions previously identified by Wang et al. [5], which were experimentally shown to impair viral entry upon single-residue substitution, and five novel interactions are proposed for future experimental studies with our active collaborators in Pharmacology. These computational findings provide high-resolution structural and dynamic validation of the critical RBD-DPP4 interface [27,28]. Targeting these specific interactions offers a clear structure-based pathway to disrupt viral entry, thereby generating valuable molecular blueprints for future experimental validation and translational drug discovery efforts against MERS-CoV.

## 2. Results and Discussion

### 2.1. Protein Structure Analysis

**Figure 1:**
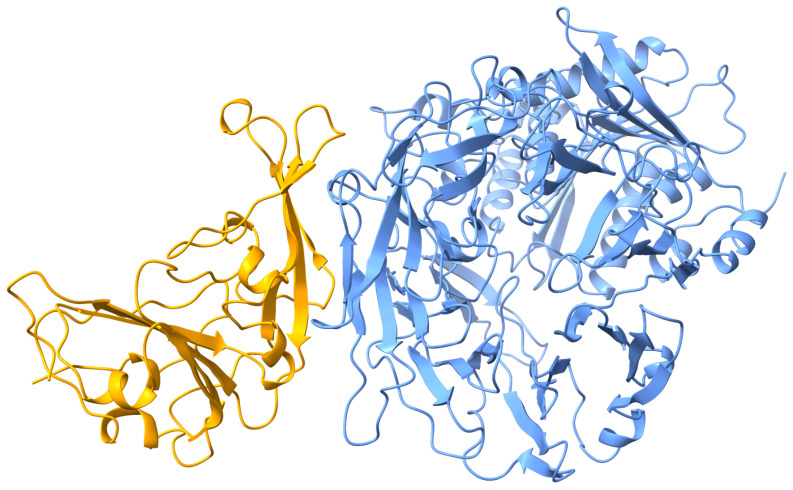
The three-dimensional protein structure of MERS-CoV RBD (orange) complexed with human DPP4 protein (blue) with PDBID 4L72 [5].

The structural integrity and key interaction sites of the MERS-CoV RBD-DPP4 complex [5] were initially assessed using the high-resolution crystal structure obtained from the Protein Data Bank [29] (PDB ID: 4L72 [5]; Ministry of Education Key Laboratory of Protein Science, Center for Structural Biology, School of Life Sciences, Tsinghua University, Beijing 100084, China). Visualization and initial structural alignment were performed using ChimeraX (1.9) [25]. To clearly demarcate the components for analysis, the viral RBD and the host DPP4 receptor were rendered in distinct colors (Figure 1). This initial characterization facilitated the spatial identification of the binding interface prior to dynamic simulation and subsequent molecular analysis.

### 2.2. Hydrogen Bond Analysis

**Figure 2:**
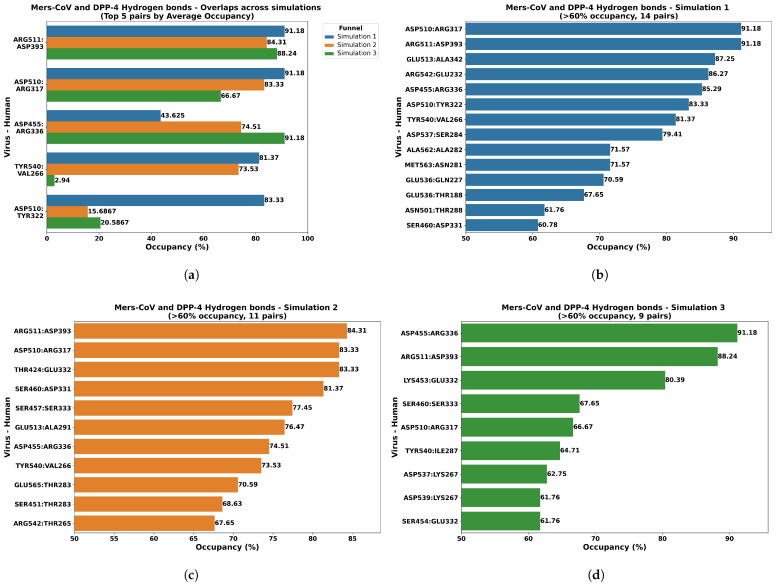
Hydrogen bond occupancy analysis of residues between MERS-CoV RBD and human DPP4 with the average occupancy overlaps (**a**) across the three simulations (**b**–**d**). The residual pair with hydrogen bonds > 60% occupancy were considered for the analysis. The title of each graph shows the number of hydrogen bonds formed during each simulation between the virus and human receptor. Each bar represents the occupancy rate of residual pairs in the order of virus to human receptor interaction, where the donor and acceptor build a pair or vice versa based on the amino–amino interactions. The x-axis represents the occupany percentage of residual pairs, and the y-axis represents the residual pairs.

The MD simulation trajectories were analyzed to quantify the hydrogen bonds forming at the MERS-CoV RBD-DPP4 interface, interactions essential for complex stabilization [18]. Considering only hydrogen bonds with an occupancy rate of at least 60% across the 1000 ns (nanoseconds) trajectory, we identified a consistent network of interactions across three independent simulation runs (Figure 2). The number of stable hydrogen bonds residue pairs identified was consistent across the three independent runs (14, 11, and 9 pairs). The maximum occupancy observed in each run was 91.18%, 84.31%, and 91.18%, respectively (Figure 2b–d). Averaged across all three independent simulation runs, the most frequent and stable hydrogen bonds were identified between virus and human proteins, such as Arg511 and Asp393, Asp510 and Arg317, and Asp455 and Arg336 (Figure 2a). These high-occupancy interactions strongly confirm that hydrogen bonding is a significant structural component mediating the stability of this key protein–protein interface.

A comparative analysis of the stable hydrogen bond network revealed a notable pattern in the donor–acceptor pairings: acidic Aspartic (Asp) residues from the viral RBD frequently formed hydrogen bonds with basic Arginine (Arg) residues on the human DPP4 receptor. This observation is highly significant as it reinforces the findings from previous studies, which indicated the critical role of charge–charge interactions in stabilizing the MERS-CoV S1 RBD-DPP4 complex [7].

### 2.3. Root Mean Squared Deviation (RMSD) Analysis

**Figure 3:**
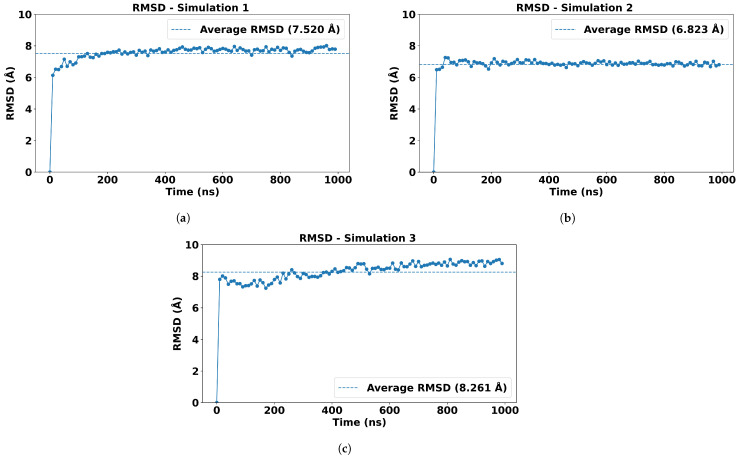
Root mean squared deviation (RMSD) analysis across independent simulations (**a**–**c**). The x-axis represents the simulation frame number, while the y-axis indicates the RMSD in Å. The dotted line indicates the average RMSD values to highlight overall trends throughout the simulation.

We further investigated the stability and conformational changes of the molecular complex through analyzing the Root Mean Sqaured Deviation (RMSD) [30] values, which is a widely used metric in MD simulations to quantify the structural deviation of a biomolecule over time relative to a reference structure. In this study, we analyzed the RMSD of protein backbone atoms ($C_{\alpha }$) across simulation frames using a custom Python script built with the MDAnalysis [31] library. The average RMSD for the MERS-CoV RBD-DPP4 complex for each independent simulation was found to be approximately 7.5 Å, 6.8 Å, and 8.2 Å, respectively (Figure 3), suggesting the overall complex maintains structural integrity, with some conformational flexibility existing within the complex over the simulation time [7].

### 2.4. Salt Bridge Analysis

**Figure 4:**
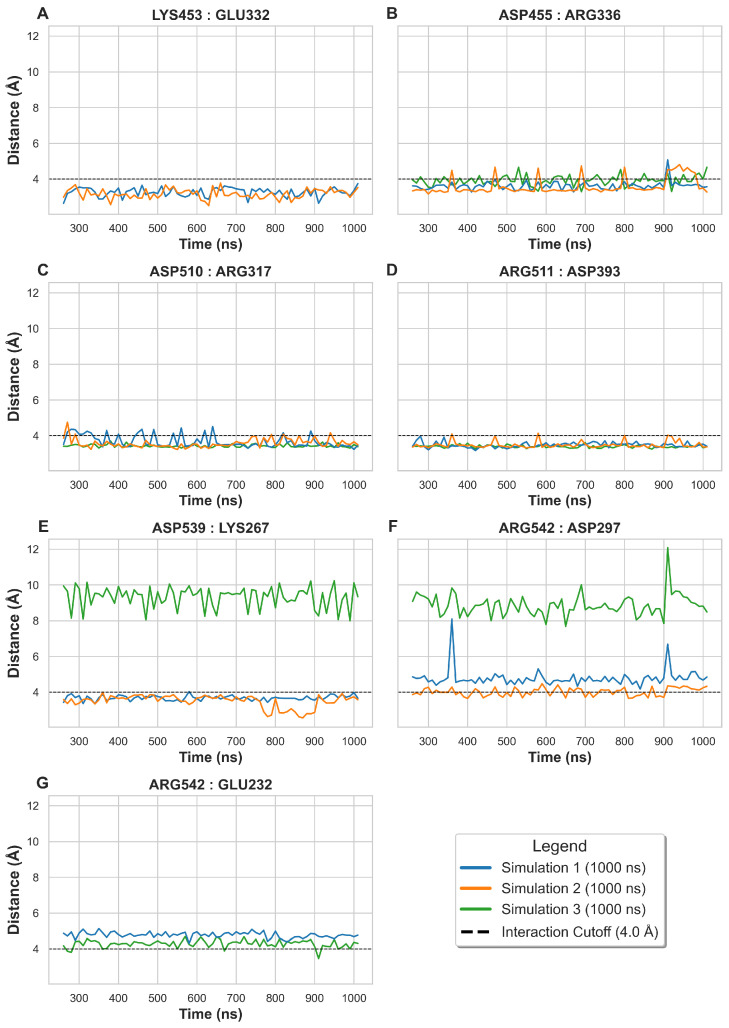
The salt bridges and distance analysis between MERS-CoV RBD and DPP4 proteins. The plots show seven critical inter-chain residue pairs (**A**–**G**) across the simulations, where each colored line represents an independent simulation replicate (Simulation 1, 2, or 3). The analysis begins at 50 ns to exclude initial spikes. The title of each chart depicts the residues in order of virus to human. The x-axis shows the simulation time, and the y-axis shows the inter-atomic distances between the residues over the time. A reference line is drawn from the y-axis at 4 Å to differentiate the strong and weak salt bridges.

The stability conferred by electrostatic interactions at the interface was quantified by analyzing salt bridges formed between the MERS-CoV RBD and human DPP4. A strong salt bridge was defined by the inter-atomic distance between charged centers consistently remaining below 4 Å. Our analysis identified seven critical residue pairs (Figure 4) that met this criterion in at least one frame across all simulation runs.

The seven pairs exhibited three distinct charge combinations. Two strong and highly consistent pairs involved the viral Aspartic acid (Asp) and host Arginine (Arg) residues: Asp455 with Arg336 (B) and Asp510 with Arg317 (C). Three pairs involved the reverse interaction or Glutamic acid (Glu): Arg511 with Asp393 (D), Arg542 with Asp297 (F), and Arg542 with Glu232 (G). The remaining two pairs were formed between Lysine (Lys) and Glu: Lys453 with Glu332 (A) and Asp539 with Lys267 (E). The consistent involvement of Aspartic acid and Arginine in forming these pivotal electrostatic contacts reinforces the patterns observed in the hydrogen bond analysis, collectively defining the essential interactions for viral–host recognition, which can be therapeutically targeted to inhibit viral entry and interference [18].

**Figure 5:**
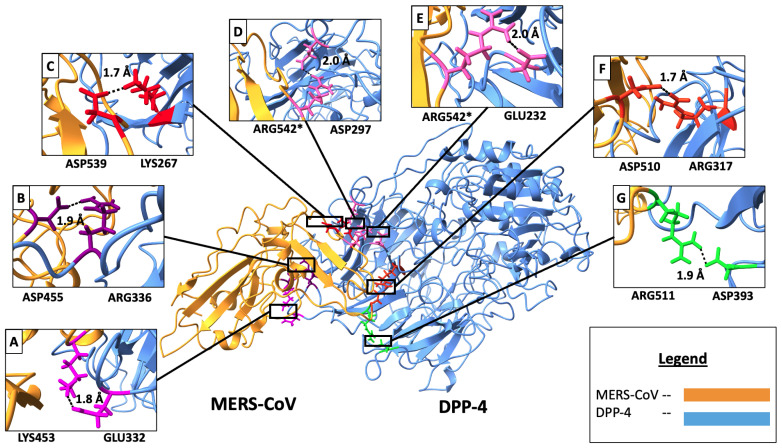
Salt bridge residue pairs highlighted over the 80th frame of the second simulation results. The distances shown are average inter-residue distances, calculated across the 1000 ns of triplicate simulations. The structure in the middle of the figure is the MERS-CoV RBD (orange) and DPP4 (blue) complex with key residues highlighted in different colors. The residue pairs are in the order of virus to human interaction: (**A**) Lys453-Glu332 (magenta); (**B**) Asp455-Arg336 (purple); (**C**) Asp539-Lys267 (red); (**D**) Arg542-Asp297 (pink); (**E**) Arg542-Glu232 (pink); (**F**) Asp510-Arg317 (orange red); (**G**) Arg511-Asp393 (green). The residue marked with an asterisk (*) indicates that it occurs more than once by forming pairs with different host or receptor residues.

The distances and specific pairs are further visualized in Figure 4, while Figure 5 maps their location onto the three-dimensional protein structure (RBD in orange; DPP4 in light blue). This spatial representation highlights the specific target amino acids within the interface, emphasizing the intermolecular distances that are crucial for successful viral entry and represent highly viable targets for therapeutic intervention.

The average inter-molecular distances for these identified salt bridge residue pairs, calculated across the three independent simulations, are summarized in Table 1, providing a quantitative measure of their stability. Residue pairs with average distances less than or near 4 Å were confirmed as the most critical salt bridges for the stability of the MERS-CoV RBD-DPP4 complex.

### 2.5. Binding Affinity Predictions

**Figure 6:**
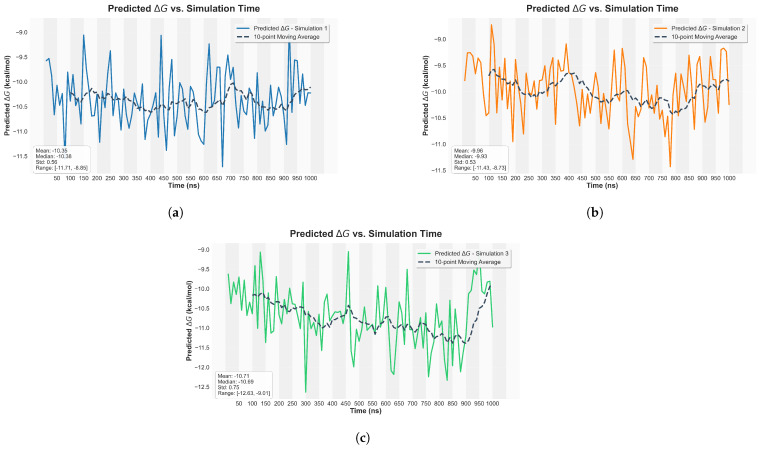
Binding affinity predictions represented as Gibbs free energy (ΔG) over the simulation time for MERS-CoV RBD and human DPP4 complex across triplicate simulations (**a**–**c**). The x-axis represents the simulation time in nanoseconds (ns), while the y-axis indicates the predicted Gibbs free energy in kcal/mol. The dotted line indicates the 10-point moving average to smooth out fluctuations and highlight overall trends throughout the simulation.

The stability of the MERS-CoV RBD-DPP4 complex was quantitatively characterized by calculating binding affinity predictions directly from the resulting MD trajectories, providing a distinct measure of the interaction strength. As detailed in Section 2.5, the predictions were generated using our established deep learning framework [26] and visualized as line plots showing the distribution of predicted Gibbs free energies (ΔG) over the simulation time (Figure 6). The calculated ΔG values consistently fell within the highly favorable range of −8 to −12 kcal/mol, indicating a thermodynamically stable and strong interaction between the viral RBD and the host DPP4 receptor throughout the dynamic simulation period [18]. These binding energy values are highly consistent with the strong interaction experimentally observed between the MERS-CoV RBD and DPP4, which is essential for viral entry into host cells [19]. The robust thermodynamic insights derived from this analysis support the previous analysis of the hydrogen bonding and salt bridge interactions.

## 3. Materials and Methods

The computational approach for investigating the complex interactions between MERS-CoV and human DPP4 are involved in several key stages, beginning with the acquisition of high-resolution structural data for both the viral RBD and the host receptor [21]. Subsequently, MD simulations were employed to model the dynamics of the complex over time, allowing for an in-depth and time-resolved analysis of interface stability and conformational changes [32].

### 3.1. Preparing Protein Structures

The three-dimensional structure of the MERS-CoV RBD complexed with human DPP4 was obtained from the Protein Data Bank (PDB) [29] using the PDB identification code (PDBID) 4L72 [5]. This crystal structure helps to establish initial atomic coordinates, providing a foundational input for subsequent computational analyses for protein–protein interaction studies, including molecular docking and molecular dynamics simulations [7]. The structural refinement process involved several critical steps: First was removing all heteroatoms from the main complex, as the focus was strictly on the protein-protein interactions [33]. Second, the polar hydrogen atoms were added to the protein structures to ensure balanced charge distributions and accurate hydrogen bonding networks during the subsequent MD simulations [34]. The integrity of the refined structure was initially assessed and visualized using UCSF ChimeraX [25]. This initial visualization confirmed the structural quality and ensured the protein complex was correctly prepared for energy minimization and subsequent MD simulation protocols.

### 3.2. Molecular Dynamics Simulation

The molecular dynamics simulation was performed using the OpenMM simulation toolkit, a high-performance computing library, under a GPU-accelerated environment [35]. This setup enabled the efficient computation of atomic interactions and trajectories over extended timeframes, which is crucial for capturing biologically relevant conformational changes [36]. The force field chosen for the simulation was AMBER14-ALL [37], and water molecules were modeled using the TIP3PFB model [38], which provides a balance between computational efficiency and accuracy for biomolecular systems. The environment to run the simulations was maintained with physiological temperature (310 K) and atmospheric pressure, allowing for the observation of protein dynamics under conditions closely mimicking the physiological context [39]. The initial velocities with respect to the temperature and energy minimization were configured to ensure proper thermalization and removal of any steric effects, before the production simulation run [40].

The simulation was conducted for a total duration of 1000 ns (nanoseconds), with trajectories extracted every 10 ns to allow for detailed analysis of the molecular evolution, similar to the architecture in Gyebi’s research [41]. To confirm the conformational stability and ensure the reproducibility of the observed molecular interactions, triplicate simulations were conducted, where each replicate was initialized with the same system parameters [42]. This rigorous iterative approach to simulation, coupled with comprehensive structural analysis, enabled the robust characterization of key residue interactions within the MERS-CoV RBD-DPP4 complex [21,43,44].

### 3.3. Salt Bridge and Hydrogen Bond Analysis

Following the completion of the MD simulations, the resulting trajectories were utilized to extract the data on salt bridges and hydrogen bonds using the Visual Molecular Dynamics (VMD) tool (1.9) [45]. For hydrogen bond analysis, we established strict criteria consistent with molecular modeling standards: a maximum donor–acceptor distance of 4.0 Å and an angle cutoff of 20 degrees. Interactions with an occupancy rate exceeding 60% over the total simulation time were considered pivotal for defining complex stability [21,24]. Similarly, salt bridges were identified based on a maximum distance threshold of 4.0 Å between the charged centers of acidic and basic residues, focusing strictly on inter-chain interactions to isolate contributions to the protein–protein interface.

### 3.4. Visualizing the Salt Bridge and Hydrogen Bonds

The identified salt bridges and hydrogen bonds were visualized using the Seaborn Python library [46]. The salt bridge data were represented as line plots, tracking the average distance between charged residues across triplicate simulations where the mean distance was below 6 Å, using 4 Å as a reference line for defining stable interactions. For hydrogen bonds, horizontal bar graphs were generated for each simulation, where each bar depicts the occupancy of a hydrogen bond, emphasizing interactions with an occupancy higher than 60%. The visualization software UCSF ChimeraX (1.9) [25] was employed for highlighting the key residues obtained from these analyses and mapping them onto the three-dimensional protein structures [24,47,48]. This approach provided a comprehensive spatially resolved understanding of the functional significance of the critical contact sites.

### 3.5. Computing Resources

All simulations and analyses were performed on HPC clusters equipped with NVIDIA GPUs [49]. Specifically, we utilized NSF ACCESS resources, leveraging the Delta GPU clusters at the National Center for Supercomputing Applications on the University of Illinois Urbana-Champaign campus (https://delta.ncsa.illinois.edu/) [50]. This dedicated computational power was essential for the acceleration of the complex MD calculations. The total computational time required was approximately 42 h per simulation when utilizing four NVIDIA A100 GPUs [51]. This highly efficient approach achieved a 10-fold performance increase compared to traditional CPU-based systems [52], thereby facilitating the rapid and thorough exploration of the MERS-CoV RBD-DPP4 interface and the reliable identification of critical contact residues.

### 3.6. Binding Affinity Prediction

To quantitatively characterize the thermodynamic stability of the MERS-CoV RBD-DPP4 complex, binding affinities were calculated using our previously established deep learning framework developed for predicting protein–protein interactions [26,53]. This framework leverages a 3D Convolutional Neural Network (CNN) [54] trained on the PDBbind v.2020 dataset [55]. It contains 2852 protein–protein complexes. Our studies will focus on dissociation constant ($K_{d}$) with further filtering performed based on its precision. Each complex has four levels of accuracy for $K_{d}$: exact values (=), approximate values (∼), and upper or lower bounds (< or >). To ensure the quality and reliability of our dataset, we selected complexes with precisely (=) or approximately (∼) reported $K_{d}$ values. This selection creates a final curated dataset of 2588 protein–protein complexes.

The model takes 3D structure coordinates extracted from the MD trajectories as input, from which three key features are generated for prediction: 1. 3D volumetric representation—A 3D volumetric representation is created by mapping the protein structure onto a fixed-size 3D grid. The protein’s Cα coordinates are first centered around the origin. Each Cα atom is then assigned to a specific voxel within the grid. The grid contains four feature channels: (1) a density value of 1.0, (2) a hydrophobicity score based on the Kyte–Doolittle scale, (3) the residue’s formal charge at physiological pH, and (4) its normalized van der Waals volume. Finally, a Gaussian filter with a sigma of 0.5 is applied to each channel to create a smoother and more continuous representation that accounts for the spatial influence of neighboring atoms. 2. Contact Map—A binary matrix representing residue proximity, which is derived from the pairwise Euclidean distance matrix of all Cα atoms. An entry in the map is set to 1 if the distance between the corresponding residues is less than a specified threshold (8.0 Å), and it is 0 otherwise. 3. Distance Map—A continuous-value matrix containing the normalized pairwise distances between Cα atoms. Distances are scaled by a maximum value (20.0 Å) and clipped to a [0, 1] range. Once three features are generated, they will go through multiple 3D CNN layers and global average pooling. The outputs will be concatenated, and a final dense layer is used to output the final predicted values.

The model outputs the predicted dissociation constant ($K_{d}$) for each frame from the simulation trajectory. The $K_{d}$ is the equilibrium ratio between the unbound and bound states and serves as a direct measure of the binding strength, where a lower $K_{d}$ signifies stronger affinity [26]. From $K_{d}$, the logarithmic binding affinity, $pK_{d}$ was calculated, where $pK_{d}=-\log_{10}\left(K_{d}\right)$, and then the Gibbs free energy was computed using the thermodynamic equation (ΔG), $\Delta G=RT\ln(K_{d})$. The final results are visualized by plotting the distribution of predicted Gibbs free energies over the simulation trajectory, allowing for a statistical assessment of the binding stability.

The model was further validated by comparing its performance with Prodigy, a well-known tool for predicting protein–protein binding affinities [56,57]. The model’s performance was evaluated using a test set of 259 protein complexes, ensuring its reliability and accuracy in predicting binding affinities. The use of this deep learning framework allowed us to quantitatively assess the thermodynamic stability of the MERS-CoV RBD-DPP4 complex over time, providing a robust foundation for structure-based drug design efforts against MERS-CoV.

As shown in Table 2, Prodigy failed 3 of the 259 predictions due to the non-standard amino acids, such as the unknown amino acid UNK, in the testing data. For the remaining 256 proteins, Prodigy likely failed the predictions, as the MAE for ΔG is 18.05. This is because the testing data contain diverse protein complexes, some with more than two chains. When the testing data are filtered to include only proteins with two chains (75 proteins), Prodigy predicts the results better, with an MAE of 2.78 and an RMSE of 3.68. While, Prodigy [56,57] reports an RMSE of 1.89 kcal/mol on its curated dataset, on our independent test set (restricted to two-chain PDBs), its RMSE increases to 3.68 kcal/mol, with very low correlations (*r* = 0.12). In contrast, even our Light V1 CNN model, which uses only 1.5 GB of GPU memory for inference, achieves more accurate and generalizable predictions. With either the full 259 testing data or the reduced 75 testing data, it achieves an MAE of 1.97. All other metrics remain largely unchanged. It can also handle diverse protein complexes with unknown amino acids and any number of chains. This demonstrates both efficiency and robustness of the model. More details on the framework is published in a separate study [53].

## 4. Conclusions

Our simulation studies revealed detailed molecular interactions governing the binding of MERS-CoV to human DPP4, specifically identifying the hydrogen bonds and salt bridges that stabilize the complex. Specifically, the residues such as Asp455, Asp510, and Arg511 of the MERS-CoV RBD and Arg336, Arg317, and Asp393 of human DPP4 are responsible for mediating strong electrostatic and hydrogen bonding interactions, respectively.

Based on the salt bridge and hydrogen bond analyses, we identified seven target residues, where two of the pairs matched with previous findings, and the additional five residual pairs contributed to future experimental studies in understanding the viral entry mechanism and the potential targets for therapeutic intervention. The top three hydrogen bonds were based on high occupancies across all simulations, Asp455-Arg336, Asp510-Arg317, and Arg511-Asp393. Furthermore, the top three salt bridges based on consistent strong interactions across all simulations were Asp455-Arg336, Asp510-Arg317, and Arg511-Asp393. This congruence strongly underscores the critical overlapping functional role of these three residue pairs in anchoring and stabilizing the MERS-CoV RBD-DPP4 complex. The prominence of Aspartic acid and Arginine residues in forming these stable electrostatic interactions suggests that disrupting these interactions can lead to a potential therapeutic strategy to inhibit viral entry.

The current computational work provides a robust foundation for structure-based drug design efforts against MERS-CoV by significantly advancing the molecular understanding of RBD-DPP4 interactions. This study emphasizes the role of computational biology in accelerating the drug discovery process and translating fundamental molecular findings into potential therapeutic applications against emerging coronaviruses. However, to fully realize the translational potential of these findings, the structural insights must be placed within the complex biological and evolutionary landscape. Single-cell and cell subpopulation detection analyses are essential for resolving cell-specific variations in DPP4 expression across diverse host tissues [58], using tools like PHet [59]. This resolution dictates viral tropism and facilitates the design of effective drug delivery strategies.

By computationally monitoring emerging MERS-CoV subtypes [60] and comparing them against our identified contact residues (such as the Asp455-Arg336 salt bridge), we can assess drug efficacy based on residue conservation and preemptively identify genetic changes that confer viral escape or resistance. In summary, this computational work elucidates the molecular interactions between the MERS-CoV RBD and DPP4 interactions, providing a structural foundation that empowers wet lab researchers to prioritize targeted experimental efforts in the design of novel inhibitors.

## Figures and Tables

**Table 1:** Summary of key residue pairs and its average inter-molecular residue distances from salt bridge analysis across all simulations. The residue pairs marked with an asterisk (*) indicate the presence of residue pair in two out of the three performed simulations.

MERS-CoV Residues	DPP4 Residues	Average Inter-Molecular Residue Distances (Å)
LYS453	GLU332	3.70 *
ASP455	ARG336	3.74
ASP510	ARG317	3.56
ARG511	ASP393	3.63
ASP539	LYS267	5.43
ARG542	ASP297	5.96
ARG542	GLU232	4.77 *

**Table 2:** Performance comparison of Prodigy and Light V1 models for ΔG prediction on all-chain and two-chain protein complex datasets.

Model (Target: ΔG)	Error Metrics				Correlation Coefficients	
	MAE	MSE	RMSE	MAPE	Pearson (*r*)	Spearman (ρ)
**Prodigy** All (259 samples, 3 failed)	18.05	1207.12	34.74	2.05	0.141	0.1319
**Prodigy** Two-chain (75 samples)	2.79	13.58	3.68	0.485	0.1201	0.1283
**Light V1 (ours)** All (259 samples)	1.97	5.83	2.41	0.256	0.2936	0.2644
**Light V1 (ours)** Two-chain (75 samples)	1.9684	6.21	2.49	0.310	0.3149	0.2382

The model names are highlighted in bold for readability. MAE: mean absolute error; MSE: mean squared error; RMSE: root mean squared error; MAPE: mean absolute percentage error.

## Data Availability

The data were obtained from the Protein Data Bank (https://www.rcsb.org), which is open to the public. The source code and simulation results supporting this study are publicly available on GitHub (https://github.com/prad101/mers-md-simulation/tree/main/results, accessed on 1 December 2025).
